# Efficacy of Biomimetic Hydroxyapatite in the Treatment of Extrinsic Dental Stains in Smokers and Non-Smokers

**DOI:** 10.3390/ma18112441

**Published:** 2025-05-23

**Authors:** Sarkis Sozkes, Maria Chomyszyn-Gajewska, Agata Dudzik, Iwona Olszewska-Czyz

**Affiliations:** 1 Biomaterials Department, Biomedical Engineering Corlu Faculty, Tekirdag Namik Kemal University, 59860 Tekirdag, Turkey; 2 Department of Bioengineering, Civil Engineering and Environmental Engineering, U.A. Whitaker College of Engineering, Florida Gulf Coast University, Fort Myers, FL 33907, USA; 3 Periodontology, Prophylaxis and Oral Pathology Department, Medical Faculty, Jagiellonian University, 31155 Krakow, Poland; mdgajews@cyf-kr.edu.pl (M.C.-G.); agata.skrzypek@uj.edu.pl (A.D.); iwona.olszewska-czyz@uj.edu.pl (I.O.-C.)

**Keywords:** hydroxyapatite, tooth staining, smokers, nicotine, biofilm, dentifrice

## Abstract

Smoking is a major risk factor for a variety of oral diseases. In particular, smoking-induced dental stains have been shown to be more refractory than those in non-smokers. Hydroxyapatite (HAP) is a biomimetic material that has been shown to be helpful in many oral health applications; however, its efficacy in stain removal in smokers and non-smokers is uncertain. To compare the effects of HAP toothpaste on the removal/control of extrinsic tooth discoloration in smokers and non-smokers. The secondary goal was to compare smokers and non-smokers in terms of staining extent and response to HAP. A total of 100 adults (50 smokers, 50 non-smokers) who met the inclusion and exclusion criteria were invited to participate in the study. At baseline and 2 weeks after the intervention, the same examiner performed clinical observations, including measurements of anterior tooth stain using the approximal plaque index (API) and the Lobene stain index. Adverse events and any changes in general health conditions of the patients were monitored. Comparisons of indices at baseline and post-intervention yielded statistically significant differences. In non-smokers, the median API (IQR) at baseline was 32.5 (19.0, 63.0) which decreased to 16.5 (7.0, 42.0) after the intervention (*p* < 0.001). The median Lobene stain index (extension) at baseline and after the intervention was 0.9 (0.5, 1.3) and 0.3 (0.2, 0.7), respectively (*p* < 0.001). In smokers, the median API at baseline (IQR) was 46.0 (30.0, 86.0), which decreased to 23.0 (7.0, 43.0) (*p* < 0.001) post-intervention. Lobene stain indices were lower after intervention in all groups than at baseline (all, *p* < 0.001), and the magnitude of reduction was more prominent in the smoker group. This study demonstrates that 2-week use of a toothpaste containing HAP can effectively reduce extrinsic tooth stains in smokers and non-smokers.

## 1. Introduction

Smoking is a major risk factor for a variety of oral diseases. It may cause oral mucosal sores, oral cancer, periodontal disease, and tooth loss in the oral cavity [1,2,3,4,5]. However, tooth color is probably the most apparent and immediate dental manifestation of smoking observed in the general population. The teeth of smokers are more prone to developing tobacco stains, which may range in color from yellow to brown to dark brown to black, with the intensity of the stain dependent on the length and frequency of smoking [6,7,8].

In an interesting study, the efficacy of nicotine replacement gum in stain reduction and shade lightening has been demonstrated [9]. However, it may be noted that, although nicotine is colorless on its own, it becomes yellow when it comes into contact with air. As a consequence, tooth darkening may have a negative impact on an individual’s look, which can lead to a social disadvantage for smokers [10,11]. Compared to non-smokers, smokers reported having substantially higher moderate and severe tooth discoloration levels in a national survey in the United Kingdom [12]. However, despite the vital importance of oral health, the degree to which smoking affects dental aesthetics and the efficacy of treatment methods has not been well studied.

Tooth stains can be classified according to the location as intrinsic or extrinsic [13]. Extrinsic dental stains are bonded to proteinaceous compounds of the salivary enamel pellicle or the biofilm. Staining compounds come from diets and habits (e.g., smoking and chewing tobacco). The bacterial biofilm and extrinsic staining can be controlled by mechanical means (e.g., tooth brushing, flossing, and others). Mechanical biofilm removal can be supported by antibacterial agents (e.g., toothpastes, mouthwashes) [14,15,16,17].

Dentin is the innermost layer of the tooth and is a bone-like biocomposite rich in proteins composed mainly of hydroxyapatite (HAP), with proteins (primarily collagen) and water accounting for the remainder [18]. The enamel, the outer layer of a tooth, is a highly mineralized tissue that contains approximately 97% HAP in the form of micrometer-long needles that create a complex hierarchical structured microstructure. HAP has a crystalline structure, typical of ceramics [19,20]. Properties such as having high melting point, being brittle/hard and biocompatible, and being typically synthesized or processed through ceramic methods, such as sintering or sol–gel processes, collectively confirm that HAP is a ceramic, more specifically, a bioceramic, and is widely used in dentistry and orthopedics due to its structural and biological properties [21,22,23,24]. Its hardness and fracture toughness are due to a complex entanglement of HAP needles linked by an organic protein phase.

A substantial amount of research has been carried out in order to develop a safe and effective formulation for tooth stain removal and prevention. An important function of dentifrice formulation is to control extrinsic dental stain formation [25]. Most dentifrices deliver this function by incorporating dental-grade particulate abrasives and using a physical mode of action to remove stains and prevent stain build-up during tooth brushing. Stain removal from tooth surfaces by tooth brushing can also be achieved by various chemical approaches, such as surfactant incorporation, to dislodge and solubilize stains [26,27,28]. Frequently used antibacterial agents are chlorhexidine, stannous salts, quaternary ammonium salts, and others [29,30,31,32,33]. However, daily use of products with some of these antimicrobials might lead to unwanted side effects. Consequently, dental research focuses on new oral biofilm management approaches [29,31,32].

HAP has been widely used for biomedical applications such as bone, cementum, and implant coatings for many years [34,35]. Studies show its effectiveness in reducing dentin hypersensitivity and remineralizing enamel and dentin [36,37,38]. In situ studies show remarkable effects of HAP particles, which reduce the initial bacterial colonization on enamel and oral surfaces. In addition, oral care products with HAP improve periodontal health under in vivo conditions [39,40].

In contrast to frequently used antibacterial agents for biofilm control, HAP particles in oral care products lead to a reduction in bacterial attachment to enamel surfaces without unwanted side effects such as tooth discoloration [36,38]. Furthermore, antibacterial agents could lead to dysbiosis of oral ecology, which, in contrast, is not observed in the case of biomimetics [41,42]. Although HAP molecules have been less well analyzed than commonly used antibacterial agents, they are a promising alternative for oral biofilm management [31,32].

The research objective of this observational study is to evaluate the effectiveness of a HAP dentifrice formulation in the management of extrinsic dental stain among smokers and non-smokers.

## 2. Materials and Methods

### 2.1. Trial Design

The trial was a single-center, 2-week observational study that enrolled participants from the Department of Periodontology of Jagiellonian University. The study was performed in accordance with the Helsinki Declaration of 2008. All participants gave their informed consent to participate in this observational study, and official approval of the study protocol was obtained from the Jagiellonian University Ethics Committee (No: 1226120292015, approval date: 30 April 2015). Participants were enrolled during periodontal care visits. Before and after the study, there were no changes in eligibility criteria and outcomes.

### 2.2. Participants

One hundred generally healthy adult participants with at least 10 teeth were enrolled in the study (50 non-smokers and 50 smokers). The sample size of this research was determined in order to observe the effectiveness of whitening toothpaste in removing tooth stains. All individuals enrolled in the study reported a primary complaint of tooth stains. Subjects were recruited from patients who had no acute lesions or diseases in the oral cavity and had not had any dental treatment for at least 3 months. None of the participants had taken antibiotics, non-steroid anti-inflammatory drugs, corticosteroids, or multivitamin supplements or used mouthwash in the last 3 months. They had to be non-smokers for a minimum of 5 years or smokers (at least one cigarette per day for at least a month), free of oral cavity disorders such as caries, epithelial dysplasia, inflammatory lesions of the oral mucosa, and severe periodontitis. History of rheumatic disorders, Sjögren disorder, enteritis, asthma, and sinusitis were also excluded from the study as they can influence the quality and quantity of saliva and staining. Pregnancy was also the exclusion criterion.

### 2.3. Exclusion Criteria

The exclusion criteria included the following: (1) severe oral disease or chronic diseases; (2) advanced periodontal disease; (3) pregnant or lactating females; (4) fluorosis or tetracycline teeth; (5) wearing orthodontic bands or partial or removable dentures; (6) participating in other clinical trials; (7) receiving prophylaxis during the previous 3 months or tooth whitening treatment during the past 6 months; (8) allergic to study products.

### 2.4. Data Collection

Data were collected by trained and certified study personnel. Study personnel recorded medical history, medication use, demographic information, and lifestyle information, including sex, age, smoking habit, and oral hygiene routine. Observational data were collected at the beginning of the study and after two weeks.

### 2.5. Intervention

The patients underwent observational assessment of plaque and staining at the beginning of the study and after two weeks. After the first examination, the patients received detailed instructions on oral hygiene principles and the use of a pure HAP dentifrice formulation (powder). Powder is named Biochem, by Chema-Elektromet Spoldzielnia Pracy, ul. Przemyslowa 9, 35-105 Rzeszow, Poland. (EU Register of Cosmetic Products No: 1024279, Polish Register of Cosmetic Products: RK/46062/2002). Ingredients: Hydroxyapatite (%70), Calcium Carbonate, Sodium Lauryl Sulfate, Glycerin, Menthol, Aroma (Mint). Patients were told to use the powder with a moist toothbrush twice a day for two weeks instead of toothpaste. During that time, the study participants did not use any other oral hygiene products except tools for hygiene of interproximal surfaces (dental floss, interdental brush). After the trial, the patients were referred for a follow-up periodontal care or additional treatment as needed.

### 2.6. Adverse Events and Safety Monitoring

Oral abnormalities were evaluated at the start of the observational study (baseline) and again after two weeks (after trial completion). Rescue treatment was not required in any of the individuals. During follow-up observations at two weeks, an investigator also asked patients if they had experienced adverse events. Discomfort during brushing, bitter or altered taste, allergic responses caused by toothpaste, and any sensation of changes in oral soft tissues were considered adverse events. Any changes in the general health of the patients caused by the product were also tracked.

### 2.7. Clinical Observations

Clinical markers such as the approximal plaque index (API), the Loben index, and the extent and severity index (ESI) were evaluated for all individuals [37]. The Loben index examines tooth stains on the labial and lingual surfaces of the lower incisors and canines and on the labial surface of the upper incisors and canines [38]. Observations of the presence of tooth stains were evaluated on both the coronal and cervical sides of the tooth. The staining is rated as 0, 1, 2, or 3 depending on whether there is no staining, staining up to 1/3 of the tooth surface, staining up to 2/3 of the tooth surface, or staining more than 2/3 of the tooth surface. The intensity of the staining was assigned a value of 1, 2, or 3 based on whether the staining was fine (yellowish color), moderate (brownish color), or intense (dark brown, black color).

Average staining value = (A sum of the staining index of the 18 sites)/18

Average intensity value = (A sum of the staining intensity of the 18 sites)/18

The extent and severity index (ESI) was used to examine stains on the surfaces of the incisor teeth [39]. This index requires the division of a tooth surface into four quadrants in order to assess the staining, with each quadrant’s staining being scored using the ‘+’ sign indicating that staining is present and the ‘-’ sign indicating that staining is absent on that particular tooth surface.

        (a sum of the staining sites)

Average staining (%) ---------------------------------------- × 100

        (number of the sites assessed)

### 2.8. Statistical Analysis

Statistical analyses were performed using a Microsoft Excel spreadsheet and SPSS19.0 statistical software. A *p* value < 0.05 was considered significant. The normality of the data distribution was assessed based on a Kolmogorov–Smirnov quantitative test. Discrepancies between the evaluation of quantitative differences were assessed based on the Wilcoxon test (due to a distribution that deviated significantly from the normal distribution).

## 3. Results

**Figure 1:**
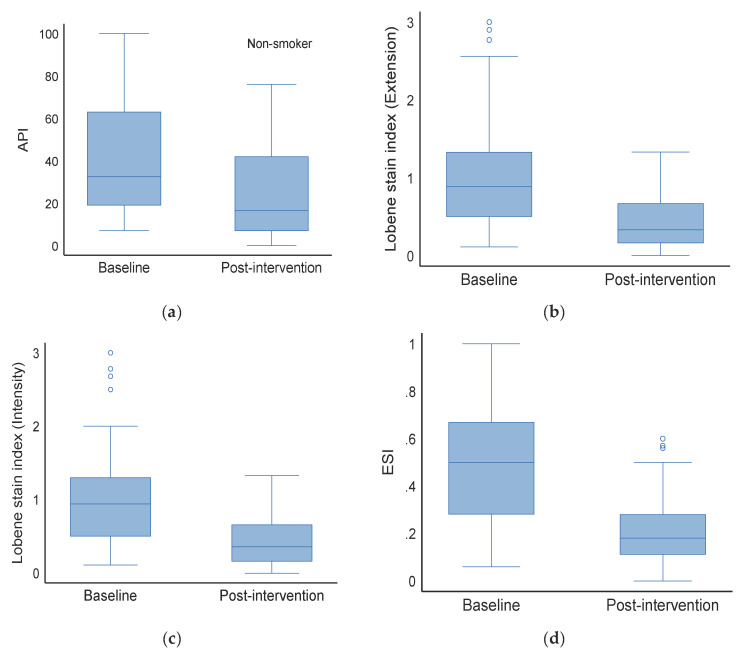
Staining indices in non-smoker group before (baseline) and after treatment (post-intervention). (**a**) API—approximal plaque index, (**b**) Lobene stain index(Extension), (**c**) Lobene stain index (Intensity), (**d**) ESI—extent and severity index.

**Figure 2:**
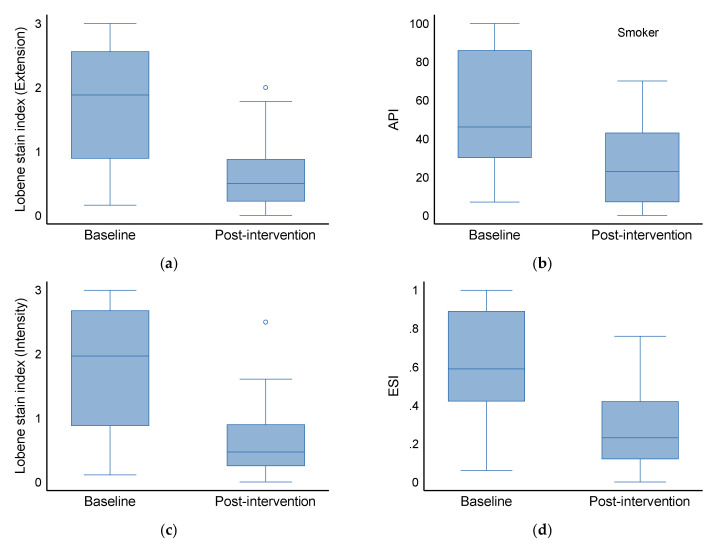
Staining indices in smoker group before (baseline) and after treatment (post-intervention). (**a**) Lobene stain index (Extension), (**b**) API—approximal plaque index, (**c**) Lobene stain index (Intensity), (**d**) ESI—extent and severity index.

At baseline, we screened patients to obtain 100 participants who met the eligibility criteria (Figure 1). The mean age of the participants was similar between the groups. Men and women were evenly represented (*p* = 0.55, Table 1). All the individuals in the smoking group maintained their smoking habits throughout the study. In particular, none of them were able to quit or restart their smoking habits. Everyone included in this observational study completed the intervention regimen (twice-daily use of a pure HAP dentifrice formulation) and was available for analysis after the completion of the trial (Figure 2). There were no dropouts or adverse events.

### 3.1. Oral Health Indices at Baseline (Smokers vs. Non-Smokers)

The non-smoking group had a median API (IQR) of 32.5 (19.0, 63.0), while the smoker group had a median API (IQR) of 46.0 (30.0, 86.0) (*p* = 0.098). In the non-smoking and smoker groups, the median Lobene stain index (Extension) was 0.9 (0.5, 1.3) and 1.9 (0.9, 2.6), respectively (*p* = 0.001). The Lobene stain index (intensity) also differed significantly between the groups [nonsmoker: 0.9 (0.5, 1.3) versus smoker: 2.0 (0.9, 2.7), *p* = 0.001]. There was a statistically significant difference between the two groups when it came to ESI [non-smokers: 0.5 (0.3, 0.7) vs. smokers: 0.5 (0.3, 0.7), *p* = 0.043] (Table 1).

### 3.2. Oral Health Indices After Trial (Smokers vs. Non-Smokers)

The non-smoking group had a median API (IQR) of 16.5 (7.0, 42.0), while the smoker group had a median API (IQR) of 23.0 (7.0, 43.0) (*p* = 0.94). In the non-smoking and smoker groups, the median Lobene stain index (extension) was 0.3 (0.2, 0.7) and 0.5 (0.2, 0.9), respectively (*p* = 0.142). The Lobene stain index (intensity) and the ESI also did not differ significantly between the groups (Table 1).

### 3.3. Effect on Non-Smokers (Baseline vs. Post-Intervention)

The median API (IQR) in the baseline group was 32.5 (19.0, 63.0), but dropped to 16.5 (7.0, 42.0) after the intervention (*p* < 0.001). The median Lobene stain index (extension) at baseline and post-intervention was 0.9 (0.5, 1.3) and 0.3 (0.2, 0.7), respectively (*p* < 0.001). The Lobene stain index (intensity) also varied substantially between the groups [Baseline: 0.9 (0.5, 1.3) vs. post-intervention: 0.4 (0.2, 0.7), *p* < 0.001]. There were also significant differences in ESI between the two groups.

### 3.4. Effect on Smokers (Baseline vs. Post-Intervention)

At baseline, the median API (IQR) was 46.0 (30.0, 86.0), which decreased to 23.0 (7.0, 43.0) (*p* < 0.001) post-intervention. In the baseline and intervention groups, the median Lobene stain index (extension) was 1.9 (0.9, 2.6) and 0.5 (0.2, 0.9), respectively (*p* < 0.001). The Lobene stain index (intensity) also differed significantly between groups [Baseline: 2.0 (0.9, 2.7) versus intervention: 0.5 (0.3, 0.9), *p* < 0.001]. There was a statistically significant difference between the two groups when it came to ESI [Baseline: 0.6 (0.4, 0.9) vs. intervention: 0.2 (0.1, 0.4), *p* < 0.001].

## 4. Discussion

Tooth plaques and stains have severe consequences for oral health, including tooth decay, gum disease, and other oral ailments [43,44,45,46]. This observational trial investigated the effectiveness of HAP in reducing tooth discoloration in smokers and non-smokers. In our study, regardless of smoking status, all major markers associated with plaque and stains were substantially reduced after HAP application. However, the magnitude of the decrease was more pronounced in smokers. In our analysis, we primarily relied on the Lobene stain index (coverage as well as intensity) that is based on a visual inspection of tooth color [47]. After 2 weeks of home use twice daily, there were statistically significant differences in the reduction in the area and intensity of the Lobene stain from baseline values. To our knowledge, this is the first research investigating the effects of HAP application on dental stains in relation to cigarette smoking status.

The acids produced by plaque bacteria lower the pH below 5.5, seep into these channels, and dissolve carbonated HAP and rods, causing the enamel to become demineralized. Intrinsic, extrinsic, and aging-related factors can be attributed to the development of dental stains [48,49]. Inadequate brushing is detrimental to oral hygiene and allows the accumulation of stained pellicle and chromogen deposits [50]. A significant amount of emphasis has been placed on developing at-home and clinical formulations for tooth whitening or staining prevention [51]. Among these developments, intracoronary bleaching is a minimally invasive, alternative treatment that addresses aesthetic concerns related to non-vital tooth discoloration [52].

Whitening formulations for home use and professional use in the dentist clinic also aimed to resolve the complications associated with dental stains. In this context, whitening is defined as any method of increasing a tooth’s optical whiteness. In a recent clinical trial, a new whitening dentifrice containing 1.0 percent hydrogen peroxide, 0.24 percent sodium fluoride, and sodium tripolyphosphate in a high-cleaning silica base was found to have significant efficacy in both tooth whitening and extrinsic stain removal and to provide a statistically significantly higher level of efficacy for both tooth whitening and extrinsic tooth stain removal [53]. Another clinical trial was recently conducted to remove tooth stains from a whitening toothpaste containing 10% high-cleansing silica, 0.5% sodium phytate, and 0.5% sodium pyrophosphate [54]. After the completion of the trial, Lobene stain-adjusted mean area and intensity scores were both found to be significantly reduced after treatment.

However, in the literature, the efficacy of whitening toothpaste is controversial [55]. Some studies found that these formulations are not practical [56]. Other clinical trials found that whitening toothpaste had a beneficial effect in reducing extrinsic tooth discoloration [57]. A systematic review included eligible clinical trials of 32 comparisons between dentifrice whitening and regular dentifrice. The difference in mean stain area between two dentifrices was found to be a reduction of <0.04 according to the original Lobene stain index in favor of the whitening dentifrice [58]. A significant amount of research has also been conducted on bleaching and in finding the methods for retaining the whitening post-bleaching [59,60,61,62]. A study also reported that chewing gum containing sodium hexametaphosphate reduced induced stain formation by 33% compared to a no-gum treatment [63], and twice-daily brushing for two weeks with a calcium carbonate/perlite toothpaste removed more extrinsic stain than a silica control toothpaste [64]. Notably, highly concentrated bleaching systems are effective as tooth-whitening systems, with few reported side effects such as transient tooth hypersensitivity [65,66,67].

Our trial provides strong evidence regarding the efficacy of HAP in stain reduction. HAP is commonly utilized for bone repair and regeneration due to its compositional resemblance to bone minerals. HAP has two types of binding sites: positively charged calcium groups (Ca^2+^) and negatively charged phosphate groups (PO_4_^3−^) [68]. These locations are scattered across the crystal structure of the matrix. HAP reduces tooth sensitivity by blocking dental tubules. HAP does this by neutralizing acids as shown in Equation (1) [68,69] caused by plaque bacteria and by providing calcium and phosphate ion building blocks, which diffuse back into the enamel to restore lost minerals. HAP also acts as a filler, repairing minute pits and fissures on the surface of the enamel, resulting in smoother, glossier enamel, with fewer sites likely to harbor plaque and stains.

Ca_5_(PO_4_)_3_(OH)  +  7H^+^  → 5Ca^2+^  +  H_2_PO_4_ + H_2_O (1)

In an oral care setting, unlike fluoride, accidental swallowing of HAP as a toothpaste ingredient is not associated with any relevant systemic health risks such as fluorosis, as HAP is the main inorganic component of all human hard tissues, such as teeth and bones [70]. In Germany, Italy, Japan, and other countries worldwide, HAP toothpastes are commercially available. However, despite promising results, clinical trials on the efficacy of HAP for stain alleviation in smokers and non-smokers have not been reported [71]. Nonetheless, an in vitro study suggested that toothpaste containing nano-HAP revealed higher remineralizing effects than amine fluoride toothpaste with bovine dentine, and comparable trends were obtained for enamel [72].

In our study, the API was not statistically different at baseline between non-smokers and smokers, notably, although the index was higher numerically. Smokers had more significant staining indices, and staining was typically bilateral, affecting several teeth in both arches. These indicators support the notion that smoking causes staining [73]. Interestingly, even in non-smokers, the extent of staining was significantly reduced after treatment, suggesting generalizable HAP application.

The stain removal efficacy of HAP can be attributed to the remineralization, abrasive effect, or chelating efficacy of the phosphate groups present in HAP [74]. Another component of HAP, Ca^2+^ ions, can also have several protective effects, particularly the release of Ca^2+^ and (hydrogen) phosphate ions upon a challenge of bacterial acid that can inhibit biofilm formation, as shown in Equation (1) [69]. Notably, the HAP toothpaste was found to be equal to the fluoride toothpaste in a recent study [73].

Smokers are known to have more resistant dental plaques because smoking affects inflammatory and immune host responses, as well as induce a negative vasoconstrictive effect, which results in a decrease in phagocyte population, number, and chimiotactisms of neutrophils [74]. In our study, a higher reduction in plaque index was found in the smoker group compared to the non-smoker group, reflecting the high potential of HAP.

The main limitation of this research may be related to the length and frequency of the intervention, as only a two-week intervention was used. Multicenter studies and studies with measurements at more time points will bolster the findings of our work. The study was not double-blind. Participants know that they are using stain-reducing treatment that can improve patient awareness of good oral hygiene practices, helping to reduce extrinsic tooth stain. In this observational study, HAP was applied by patients themselves using a toothbrush; however, the tooth brushing protocol and the type of toothbrush were also not controlled. Electric toothbrushes are known to be more efficient in plaque removal compared to manual toothbrushes; therefore, such differences among participants may induce some biases [74]. In the case of gingivitis, plaque control was widely shown to significantly improve gingival inflammation; the treatment of HAP on other oral pathologies should be performed in future studies.

## 5. Conclusions

Oral health is significantly impacted by tooth discoloration. According to this study, smokers exhibit more staining than nonsmokers. Both smokers and non-smokers experience a significant decrease in total staining after the HAP application. Periodontists should be aware of the lesser-known benefits of HAP in stain removal, especially since it is less harsh and has fewer side effects than bleaching or other therapeutic procedures. Lastly, to assess the role of HAP, multicentric studies that are long-term will be required. Oral hygiene products containing HAP may be useful for a variety of clinical oral hygiene problems due to their positive behavior in terms of remineralization and stain avoidance.

## Figures and Tables

**Table 1:** Demographic and staining indices before and after treatment.

		Non-Smoker			Smoker			Total			*p*-Value
		(N = 50)			(N = 50)			(N = 100)			
		Median	Q1	Q3	Median	Q1	Q3	Median	Q1	Q3	
**Baseline**	**Approximal plaque index (API)**	32.5	19.0	63.0	46.0	30.0	86.0	41.0	21.0	71.0	0.098
	**Lobene stain index (extension)**	0.9	0.5	1.3	1.9	0.9	2.6	1.1	0.7	2.3	0.001
	**Lobene stain index (intensity)**	0.9	0.5	1.3	2.0	0.9	2.7	1.1	0.6	2.1	0.001
	**The extent and severity index (ESI)**	0.5	0.3	0.7	0.6	0.4	0.9	0.5	0.3	0.8	0.043
**Post-intervention**	**Approximal plaque index (API)**	16.5	7.0	42.0	23.0	7.0	43.0	20.5	7.0	42.5	0.937
	**Lobene stain index (extension)**	0.3	0.2	0.7	0.5	0.2	0.9	0.4	0.2	0.9	0.142
	**Lobene stain index (intensity)**	0.4	0.2	0.7	0.5	0.3	0.9	0.4	0.2	0.8	0.149
	**The extent and severity index (ESI)**	0.2	0.1	0.3	0.2	0.1	0.4	0.2	0.1	0.3	0.091
	**Sex**										0.548
	Male	23 (46.0%)			26 (52.0%)			49 (49.0%)			
	Female	27 (54.0%)			24 (48.0%)			51 (51.0%)			

## Data Availability

The raw data supporting the conclusions of this article will be made available by the authors on request.
